# Are virtues national, supranational, or universal?

**DOI:** 10.1186/2193-1801-3-223

**Published:** 2014-05-02

**Authors:** Jan Pieter van Oudenhoven, Boele de Raad, Marieke E Timmerman, Françoise Askevis-Leherpeux, Pawel Boski, Carmen Carmona, Rajneesh Choubisa, Alejandra del Carmen Dominguez, Hege H Bye, Anastacia Kurylo, Cornelia Lahmann, Khairul Mastor, Eva Selenko, Alena Slezáčková, Ripley Smith, Linda Tip, Michelle Yik

**Affiliations:** Department of Psychology Grote Kruisstraat, University of Groningen, Groningen 2/1, Kragujevac, 9712 TS Netherlands; Université Lille Nord de France, Lille, France; Warsaw School of Social Psychology, Warsaw, Poland; Universitat de Valéncia, Av Blasco Ibáñez, Valencia, 13 46010 Spain; Birla Institute of Technology and Science, BITS, Pilani, Rajasthan 333031 India; Ibero-American University, Prolongación Paseo de la Reforma No. 880, Lomas de Santa, Fe 01219 Mexico; Department of Psychosocial Science, University of Bergen, Bergen, Norway; Centre for Intercultural Media Research, Kragujevac, Illinois USA; University of Kuala Lumpur, Kuala Lumpur, Malaysia; University of Sheffield, Western Bank, Sheffield, South Yorkshire, S10 2TN UK; Masaryk University Brno, Kamenice 126/3, 625 00 Brno, Czech Republic; Bethel University St Paul, 3900 Bethel Dr, St Paul, MN 55112 USA; University of Sussex, Brighton, BN 1 9RH UK; Hong Kong University of Science and Technology, Clear Water Bay, Kragujevac, Hong Kong

## Abstract

Many studies investigated cultural differences in values, most notably by Hofstede and Schwarz. Relatively few have focused on virtues, a related and important concept in contemporary social science. The present paper examines the similarities and differences between nations, or blocks of - culturally related - nations on the perceived importance of virtues. Adults (N = 2.809 students) from 14 countries were asked to freely mention which virtues they found important to practice in daily life, and next to rate a list of 15 virtues, which reflect the most frequently mentioned categories in The Netherlands, as found in a previous study. The 14 nations included the United States, Mexico, nine European and three Asian nations. For the free-listed virtues, we compared the top-ten lists of most frequently mentioned virtues across the nations. We used a correspondence analysis on the frequency table to assess the relationships between the virtues and nations. For the 15 virtues ratings, a MANOVA, and follow-up ANOVA’s were used to examine effects of nation, age, gender and religion. We found strong evidence for relationships between nations and blocks of culturally related nations and the importance attached to various virtues. There appear to be some country specific virtues, such as generosity in France, but also some relatively universal virtues, most notably honesty, respect, and kindness.

**Electronic supplementary material:**

The online version of this article (doi:10.1186/2193-1801-3-223) contains supplementary material, which is available to authorized users.

## Introduction

The present study reports the results of a comparison of virtues among 14 nations. We want to know to what extent virtues are universal, or rather culturally specific. The study applied both open and close-ended questions to examine contemporary virtues.

For decades psychologists have been interested in concepts related to morality. Most notable is Piaget’s (1997) and Kohlberg’s (1981) work on the development of moral judgment in children. Currently, morality has become important in several disciplines of psychology. In modern evolutionary psychology, for example, altruistic behavior is considered crucial for the cohesion of the group and consequently for the survival of the species. The psychologist Baumeister (2005) states in his book on *The Cultural Animal* that the human being is a moral animal. Most outspokenly, De Waal (2006) argues that human morality grows from our genes and that the traits that define morality — empathy, reciprocity, reconciliation, and consolation — can be observed in many animals, particularly in primates.

More than half a century ago, Erikson (1959,1982) stressed the importance of virtues; moreover, he distinguished eight virtues, or basic strengths, namely hope, will, purpose, competence, fidelity, love, care, and wisdom which according to him become respectively relevant to one of the eight stages of human development. More recently, in the last two decades, the *Positive Psychology* movement led the field to focus on human strengths and virtues. Two key publications were Seligman’s and Csikszentmihalyi’s special issue on positive psychology in the *American Psychologist* (Seligman and Csikszentmihalyi 2000) in which they state that psychology had too much focused on pathology, and should focus instead on the positive features that make life worth living; and Dahlsgaard et al. (2005) comparative study of six dominant religious and philosophical traditions around the world: Confucionism and Taoism, Buddhism and Hinduism, Athenian philosophy, Judaism, Christianity, and Islam. Dahlsgaard et al. (2005) listed many virtues they observed in the relevant texts of these various traditions, such as the Ten Commandments for Judaism, the Bhagavad Gita for Hinduism, Analects for Confucianism, Aquino’s Theologiae for Christianity, the Koran for Islam, Aristotle’s Nicomachean Ethics, and the Holy Eightfold Path for Buddhism. They were able to summarize them into a group of six core virtues: courage, justice, humanity, temperance, wisdom, and transcendence. They claim to have come up with more or less universal virtues. While Dahlsgaard et al. (2005) identified their many virtues in the texts they studied, Van Oudenhoven et al. (2012) followed a free response procedure, in which they asked teachers, politicians, students, and adults from various religious and non-religious backgrounds to generate virtues freely. The most frequently mentioned types of virtues in that study, which took place in the Netherlands, are represented in a list of 15 virtues (respect, justice, wisdom, joy, resolution, mercy, reliability, hope, courage, faith, moderation, openness, modesty, love, and helpfulness). In the present study we continue applying this free response format, and we want to investigate to which degree virtues are indeed universal, or rather national, or - possibly - supranational, i.e., related to blocks of nations.

We define virtues as “morally good traits that everyone either may possess or can learn” (Van Oudenhoven et al., 2012). In this way, they form a desirable subset of personality traits. Virtues can also be seen as a subset of values. There appears to be overlap between the virtues referred to as character strengths by Peterson and Seligman (2004) and Schwartz (1992) individual motivational values (Haslam et al. 2004). Virtues dictate how the individual ought to behave or ought to be. Values do not necessarily dictate how one ought to behave or ought to be. Examples of such values are intelligence, beauty, tradition or liberalism. Furthermore, virtues are typically more concrete than values. Because of their concrete nature, virtues may often be conveyed well (Van Tongeren, 2003).

Dahlsgaard et al. (2005) based their virtues on religious and philosophical sources. In contrast, we want to base our research of virtues on those personal characteristics that contemporary laypersons see as virtues, rather than on what their religious or spiritual affiliation tells them to do. Another difference with most of the current research on virtues is that we do not limit our research to the widely used closed-ended questionnaires. The use of closed-ended questionnaires is mentioned as one of the shortcomings of most of the research on virtues (Smith et al. 2007), because they might prevent respondents from coming up with virtues that may be important to them, but are not included in the questionnaires.

When Kohlberg (1958) developed his theory on moral reasoning of children, he did not take into account the culture of the child. However, with respect to virtues that give concrete indications for morally good behavior it is plausible, if not inevitable, that cultural differences do play a role. One may expect clear cultural differences in interpretation of - for instance - what is helpfulness or altruism, depending on whether one is living in a social welfare state or not. Haidt (2007, p. 999) argues that morality is universal, but culturally variable: “…contradictions are dissolving as research from many disciplines converges on a few shared principles, including the importance of moral intuitions, the socially functional (rather than truth-seeking) nature of moral thinking, and the coevolution of moral minds with cultural practices and institutions that create diverse moral communities.” And likewise we may expect social-ideological differences across nations. In line with this expectation of cultural differences, Smith et al. (2007) found only 10% of overlap in the frequencies with which representatives from seven different cultures freely listed specific qualities of a good person.

In view of globalization the question arises whether we must expect fundamental virtues to diverge or converge in societies that become increasingly culturally diverse. Some evidence for convergence of virtues within a multicultural society was found in a previous study (Van Oudenhoven, et al., 2012) which took place in The Netherlands among teachers, local politicians, secondary school students, and adults from various religious and non-religious backgrounds. Only minor differences were found on the importance rating of virtues between these groups. It was remarkable that the findings revealed only marginal differences in the importance ratings between Muslim respondents and non-Muslim respondents. In the second part of that study, adult respondents from Germany, Spain, and The Netherlands participated. Small differences were found between the more individualistic Germany and The Netherlands, but relatively large differences between these two countries compared with the more collectivistic Spain. German and Dutch respondents scored considerably higher than Spanish respondents, in particular on the importance ratings of openness and reliability, which may be considered important in individualistic cultures. The results suggest that national cultures may influence virtues, and that culturally resembling nations, such as Germany and the Netherlands, may also resemble each other more than culturally divergent nations.

In order to examine the relative convergence or divergence of moral values between various nations, we performed a comparative study of virtues from 14 countries that differ to various degrees in national culture. We have chosen a sample of nations from four different continents, with representatives from different religious and secular backgrounds and eleven different languages. We will examine the following questions:

1. *Which personal characteristics do contemporary laypersons see as virtues?*
2. *Do virtues considered to be important differ across national cultures?* Some evidence for national differences in virtues was found between Spanish respondents on the one hand and German and Dutch respondents on the other hand in the above mentioned study by Van Oudenhoven et al. (2012). We will conclude that they do if we find clear differences between nations in the kind and the frequencies of virtues that are freely mentioned, and if we find compelling differences between the importance ratings of the virtues across nations.
3. *Are virtues shared by nations that show resemblance with respect to language, culture and/or religious composition?* We will conclude that they are if we find overlap in the kind of virtues that are freely mentioned across the nations belonging to one cluster, and if we find relatively small or no differences between the importance ratings of virtues of the nations within a cluster. Among the 14 nations there are three cultural/linguistic clusters: A Hispanic cluster consisting of Spain and Mexico; an Anglo-Saxon cluster consisting of Great Britain and the United States; and a Germanic cluster consisting of Germany, Austria, and the Netherlands. The Netherlands may also be considered to belong to the Germanic cluster because German and Dutch are related – West-Germanic – languages. In a similar vein there may also be a Slavic cluster consisting of Poland and the Czech Republic because they have related languages. Germans and Dutch indeed seem to share virtues to some extent as was found in the study of Van Oudenhoven et al. (2012).
4. *Are there - relatively - universal virtues?* We will conclude that a virtue is - relatively - universal if the freely mentioned virtue is shared by more than half of the nations as one of the ten most frequently mentioned virtues, and if its importance ratings across nations is above average. Dahlsgaard et al. (2005) suggest that they have found evidence for a set of universal virtues.
5. *Are there nation specific virtues?* We will conclude that a virtue is nation specific if that freely mentioned virtue is exclusively mentioned among the top ten in one nation. As far as is known, there is no - clear – evidence for virtues that are considered to be important in only one nation.
6. Do virtues differ across gender, age or religion? No strong differences related to these three biographical variables were found in the Van Oudenhoven et al. (2012). Nevertheless it is important to check again whether virtues differ across these variables. Erikson (1959,1982) explicitly relates specific virtues to certain age stages. Religions differ explicitly in moral principles and practices they preach. And males and females may differ strongly in - moral – behavior.

## Method

### Respondents

Respondents (see Table 1) were 2,908 university students from 14 nations: Austria 276; Czech Republic 231; France 216; Germany 220; Hong Kong 101; India 152; Malaysia 211; Mexico146; Norway 187; Poland 163; United Kingdom 149; United States 184; Spain 202; and The Netherlands 371. The main religions were: none 41.5%; Roman Catholic 25.1%; Protestant 12.6%; Muslim 7.5%; Hindu 5.2%; Buddhist 2.4%; others 4.9%; missing 7.9%. The average age was 23.6 (SD = 7.04), and 38% were males. Most of the students were approached by the national researcher at several faculties during lecture breaks, or in recreational areas on university campuses.

**Table 1:** **Number of respondents per nation, percentages of males/females, mean age and first religion per nation**

Nation	Number of Respondents	Percentage of males	Percentage of females	Mean age	Percentage without religion	Most frequently mentioned religion
**Malaysia**	**211**	**77,9**	**22,1**	**21,26**	**1,0**	**Muslim**
**Spain**	**202**	**52,5**	**47,5**	**26,77**	**40,4**	**Catholic**
**Czech Republic**	**231**	**48,1**	**51,9**	**20,42**	**87,8**	**Catholic**
**Netherlands**	**371**	**28,5**	**71,5**	**24,87**	**69,8**	**Protestant**
**United Kingdom**	**149**	**22,1**	**77,9**	**21,96**	**73,2**	**Protestant**
**Austria**	**276**	**30,9**	**69,1**	**25,05**	**24,6**	**Catholic**
**Germany**	**220**	**44,4**	**55,6**	**30,39**	**39,8**	**Protestant**
**Poland**	**163**	**20,9**	**79,1**	**24,59**	**33,1**	**Catholic**
**Hong Kong**	**101**	**54,5**	**45,5**	**20,58**	**80,2**	**Protestant**
**India**	**152**	**69,1**	**30,9**	**22,16**	**0**	**Hindu**
**United States**	**183**	**28,4**	**71,6**	**24,65**	**11,0**	**Protestant**
**Norway**	**187**	**18,7**	**81,3**	**23,65**	**64,1**	**Protestant**
**France**	**216**	**15,7**	**84,3**	**19,48**	**48,6**	**Catholic**
**Mexico**	**149**	**39,7**	**60,3**	**20,84**	**21,4**	**Catholic**
**Total**	**2809**	**37,9**	**62,1**	**23,62**	**41,5**	**Catholic**

### Instrument

Respondents filled out a questionnaire in their native language. They started with answering a series of demographic questions on their gender, age, educational level, country of birth, maternal language, and religion or philosophy of life. Next, three open-ended questions were asked: First, they were asked to mention: “What do you find important personal characteristics which you would like to bring into practice in daily life?”. Second, they were asked: “What are bad personal characteristics to you?”. Third, they were invited to mention: “Which characteristics may, to your opinion, improve relations between different cultural groups in your country of origin?” In this study only answers to the first question were taken into consideration.

Finally they all answered the same closed-ended question. Respondents had to distribute a set of 15 virtues among five columns, three in each column according to the importance attributed to them. This way, a 5-point scale was formed from 1 (= least important) to 5 (= most important). This list of 15 virtues (respect, justice, wisdom, joy, resolution, mercy, reliability, hope, courage, faith, moderation, openness, modesty, love, and helpfulness) was developed on the basis of interviews with local spiritual leaders and surveys among a wide range of different groups of respondents in a previous study (Van Oudenhoven et al., 2012). They reflected the most frequently mentioned categories of virtues in the Netherlands. Interestingly, they include the four cardinal virtues (wisdom, justice, moderation, and courage) and the three theological virtues (faith, hope and love) which are often mentioned in the international philosophical literature on virtues. Data from the non-English-speaking countries were translated into English by researchers who had the specific national non-English language as their native language and were fluent in English (see Additional file 1 for the Questionnaire).

## Results

### Free-listed virtues

First, we consider the answers to the open-ended question: “What do you find important personal characteristics which you would like to bring into practice in daily life?”. Table 2 shows the rank orders of the ten most frequently mentioned virtues in each nation. The virtues, mentioned by at least half of the 14 nations among their top ten are: honesty (mentioned in all 14 nations), respect (11 nations), kindness (10 nations), openness (9 nations), and tolerance (8 nations). These virtues can be considered as - relatively - universal virtues.

**Table 2:** **Rank order (1–10) of the freely mentioned virtues in 14 nations, frequencies are indicated below the virtue**

Rank order Nation	1	2	3	4	5	6	7	8	9	10
**Austria**	Honesty	Helpfulness	Kindness	Reliability	Openness	Punctuality	Tolerance	Ambition	Resolution	Humour
**N = 276**	169	80	79	68	47	45	42	29	27	27
	61%	29%	29%	25%	17%	16%	15%	11%	10%	10%
**Czech Republic**	Resolution	Reliability	Courage	Wisdom	Openness	Helpfulness	Tolerance	Honesty	Respect	Punctuality
**N = 231**	58	43	42	30	27	23	22	19	19	16
	25%	19%	18%	13%	12%	10%	10%	8%	18%	7%
**France**	Generosity	Courage	Honesty	Kindness	Respect	Empathy	Patience	Altruism	Smartness	Openness
**N = 213**	55	52	52	41	38	30	28	21	20	16
	26%	24%	24%	19%	18%	14%	13%	9%	9%	%
**Great Britain**	Kindness	Honesty	Humour	Happiness	Respect	Resolution	Patience	Helpfulness	Empathy	Openness/Trust
**N = 149**	68	34	34	26	24	19	18	17	17	16
	46%	23%	23%	17%	16%	13%	12%	11%	11%	11%
**Germany**	Honesty	Openness	Reliability	Respect	Tolerance	Kindness	Helpfulness	Humour	Punctuality	Justice/Joy
**N = 220**	157	80	67	66	63	58	50	32	30	25
	71%	36%	30%	30%	29%	26%	23%	15%	14%	11%
**Hong Kong**	Honesty	Courtesy	Tolerance	Consideration	Optimism	Responsibility	Resolution	Self-confidence	Respect	Modesty
**N = 101**	30	13	12	12	11	11	11	10	10	10
	30%	13%	12%	12%	11%	11%	11%	10%	10%	10%
**India**	Diligence	Kindness	Joy	Dynamism	Humour	Love	Intelligence	Caring	Honesty	Reliability/Courage
**N = 152**	27	13	12	12	12	11	11	9	9	9
	18%	9%	8%	8%	8%	7%	7%	6%	65	6%
**Malaysia**	Honesty	Respect	Happiness	Love	Kindness	Helpfulness	Optimism	Courtesy	Responsibility	Faith
**N = 212**	35	26	23	22	21	20	15	14	14	14
	17%	12%	11%	10%	10%	9%	7%	7%	7%	14%
**Mexico**	Respect	Honesty	Happiness	Responsibility	Love	Peace	Friendship	Certainty	Tolerance	Loyalty/ Empathy
**N = 146**	54	38	31	30	26	19	19	13	12	10
	37%	26%	21%	20%	18%	13%	13%	9%	8%	7%
**Netherlands**	Respect	Kindness	Honesty	Helpfulness	Social	Openness	Joy	Tolerance	Reliability	Responsibility
**N = 660** ^**1**^	203	201	175	129	77	64	58	58	57	56
	31%	30%	27%	20%	12%	10%	9%	9%	9%	8%
**Norway**	Openness	Empathy	Kindness	Honesty	Optimism	Humour	Social	Consideration	Patience	Helpfulness/Caring
**N = 190**	72	55	52	48	44	32	31	30	23	23
	38%	29%	27%	25%	23%	17%	16%	16%	12%	12%
**Poland**	Openness	Honesty	Respect	Tolerance	Optimism	Communicativeness	Empathy	Resolution	Kindness	Humor
**N = 163**	52	31	26	24	21	19	19	18	17	17
	32%	19%	16%	15%	13%	12%	12%	11%	10%	10%
**Spain**	Respect	Love	Honesty	Friendship	Happiness	Joy	Justice	Solidarity	Tolerance	Trust
**N = 203**	86	44	38	32	23	17	15	14	13	13
	42%	22%	19%	16%	11%	8%	7%	7%	6%	6%
**United States**	Honesty	Kindness	Caring	Respect	Empathy	Patience	Openness	Modesty	Love	Humor/Diligence
**N = 191**	105	57	36	31	28	26	24	22	21	20
	55%	30%	19%	16%	15%	14%	13%	12%	11%	10%

^1^For the Netherlands, we disposed of answers to this particular question from an additional comparable group (N = 289) of higher educated respondents and added these to the sample for the current study (N = 371).

As virtues that are important in specific nations, the following are salient: Generosity (“Générosité” in French) was only mentioned in France among the top ten, where it was also the most frequently mentioned virtue. Other virtues that were mentioned in only one country in the top ten were ambition (Austria), wisdom (Czech Republic), communicativeness (Poland), dynamism (India), faith (Malaysia), and peace and certainty (Mexico). Those virtues, except for certainty (called “Seguridad” in Mexico), were also mentioned in other nations, albeit not in their top ten. We may safely say that generosity and certainty are national virtues of France and Mexico, respectively.

When we look at Table 2 we can see the Germanic cluster with Germany, Austria, and The Netherlands, combined with the Czech Republic to form a cultural block. Germany shares 8 virtues (honesty, helpfulness, kindness, reliability, openness, punctuality, tolerance and humour) out of its top 10 with Austria, 7 virtues (honesty, helpfulness, reliability, respect, openness, tolerance and punctuality) with the Czech Republic, and also 7 (honesty, helpfulness, kindness, respect, openness, reliability, and tolerance) with The Netherlands. Austria and the Czech Republic share 7 virtues. The Netherlands share 6 virtues both with Austria and the Czech Republic. There seem to be two more blocks: One block consists of the Anglo-Saxon cluster Great Britain and the United States, combined with Norway. Altogether they share 6 virtues (kindness, honesty, humour, empathy, openness and patience). The other block consists of the Hispanic cluster (Spain and Mexico) that share 6 virtues out of the top ten of most frequently mentioned virtues (respect, love, honesty, friendship, happiness and tolerance). The Slavic cluster consisting of Poland and the Czech Republic only share 5 virtues (openness, honesty, respect, tolerance, and resolution).

To further examine the associations between the freely mentioned virtues and the nations, we performed a Correspondence Analysis (CA), using the R package CA (Nenadic and Greenacre, 2007). We did so on the frequencies for each of the 40 virtues that were mentioned in at least one of the 14 nations as one of the ten virtues with the highest frequency. CA is a multivariate statistical method for exploring relationships in large data sets (Nenadic and Greenacre 2007). The relationships between the nominal variables (here: nation and virtue) are visualized in a spatial map, allowing an interpretation of their associations. In this study, a data matrix was constructed, with the 14 nations in the rows, and the 40 the virtues in the columns. The closer the nations in this space are to each other and the further they are away from the origin (0,0), the stronger the relationships between these nations.

**Figure 1:**
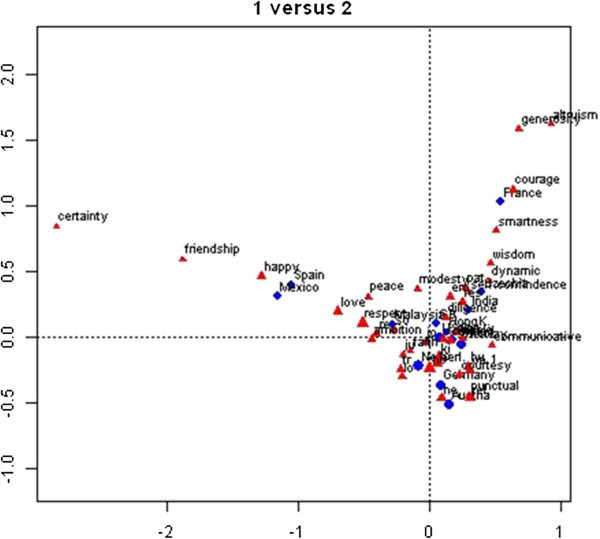
**Graphical representations of the relations of nations and virtues, as resulting from the correspondence analysis: labels of virtues close to origin are indicated with the first two letters only, to enhance readability.** The picture shows which virtues prevail in which nation, for instance happiness in Spain and Mexico.

**Figure 2:**
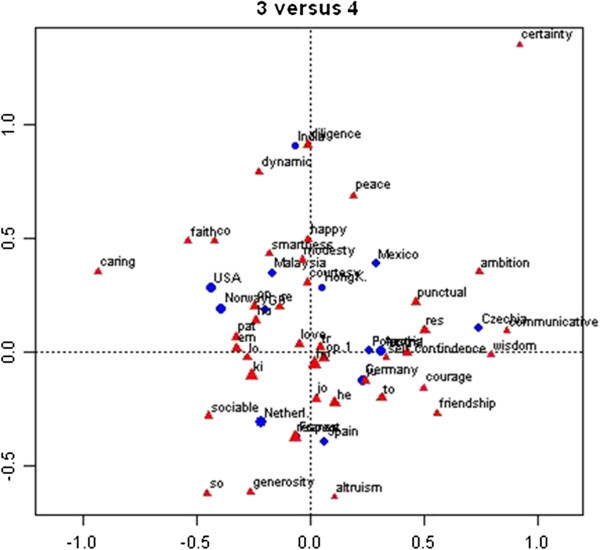
**Graphical representations of the relations of nations and virtues, as resulting from the correspondence analysis: labels of virtues close to origin are indicated with the first two letters only, to enhance readability.** The picture shows which virtues prevail in which nation, for instance wisdom in the Czech Republic.

We chose for a four-dimensional subspace in order to project the virtues onto a plane that makes easy visualization and interpretation possible, after verifying that this solution fitted the data to a reasonable extent (62.12% of the inertia explained). Figures 1 and 2 present the spatial (symmetric) maps of dimensions 1 versus 2, and of dimensions 3 versus 4, respectively. Basically, the CA enables us to show which virtues are dominant in which nations.

In Figure 1, we notice that virtues of *Goodness* (generosity, altruism, courage and smartness) are only frequently mentioned in France, and virtues of *Peace of Mind* (certainty, friendship, happiness, peace and love) in Mexico and Spain. In Figure 2 we notice that France, Spain, and the Netherlands share an interest in *Social Relations* (respect and sociable), while the United States and Norway share a preference for *Spirituality* (faith and caring). Malaysia, Hong Kong, and Mexico form a cluster regarding *Gentleness* (modesty, courtesy and happiness). India has a rather unique preference for *Action* (dynamism and diligence), and Poland and the Czech Republic for *Self-confidence* (self-confidence, wisdom and communicativeness).

### Answers to the close ended questions

The mean (and S.D.) of the importance ratings (1 = least important; 5 = most important) of the fixed set of 15 virtues per nation are presented in Table 3. The five virtues rated most important in the whole sample are: respect, love, justice, joy and reliability; the virtues rated least important are moderation, mercy, faith, modesty, and hope. A MANOVA was performed on the 15 virtue ratings with nation as independent variable. A significant effect of nation was found, *F* (15, 2781) = 31823,86; *p* < .0001. ANOVA’s showed significant effects of nation with respect to all 15 virtue ratings (all *p* < .0001). The smallest difference between the highest and the lowest mean score of the 14 nations on a virtue was .61 (for love); the largest difference between the highest and the lowest mean score of the 14 nations on a virtue was 2.97 (between Austria and Malaysia for faith). There is an average difference of 1.78 in importance ratings between the highest and lowest scores across the 14 virtues. The results clearly show national differences in virtues.

**Table 3:** **Mean (and S.D. within parentheses) ratings of 15 virtues in 14 nations (1 = least important; 5 = most important), and means and rankings across 14 nations**

Virtues	Respect	Courage	Justice	Faith	Love	Wisdom	Moderate	Joy	Open	Reliable	Resolute	Modesty	Helpful	Mercy	Hope
**Malaysia**	4.32	3.10	3.98	4.42	3.78	2.85	2.98	2.85	2.63	3.21	2.31	3.24	3.27	2.65	2.21
**N = 211**	(.94)	(1.23)	(1.08)	(1.12)	(1.17)	(1.32)	(1.28)	(1.28)	(1.33)	(1.33)	(1.27)	(1.30)	(1.20)	(1.17)	(1.26)
**Spain**	4.72	2.60	4.25	2.16	4.16	3.40	2.51	3.82	2.20	2.49	2.34	2.81	3.28	2.38	2.71
**N = 202**	(.82)	(1.15)	(1.02)	(1.32)	(1.14)	(1.07)	(1.15)	(1.07)	(1.19)	(1.15)	(1.24)	(1.20)	(1.07)	(1.20)	(1.25)
**Czechia**	3.52	2.79	3.85	1.98	4.17	3.74	1.55	3.33	2.59	3.86	2.79	2.32	3.37	2.49	2.62
**N = 231**	(1.18)	(1.34)	(1.10)	(1.30)	(1.11)	(1.25)	(.91)	(1.25)	(1.26)	(1.17)	(1.30)	(1.20)	(1.18)	(1.20)	(1.30)
**Netherlands**	4.15	2.30	3.90	2.07	4.10	3.16	2.33	3.61	3.11	4.09	2.46	2.40	3.75	2.33	2.32
**N = 371**	(1.05)	(1.13)	(1.08)	(1.49)	(1.14)	(1.24)	(1.15)	(1.22)	(1.27)	(.94)	(1.22)	(1.12)	(1.13)	(1.06)	(1.13)
**Great Britain**	4.52	3.11	3.48	2.23	4.39	3.10	1.86	3.55	2.87	3.14	1.82	2.31	3.60	2.32	2.89
**N = 149**	(.77)	(1.16)	(1.21)	(1.33)	(1.02)	(1.24)	(1.05)	(1.27)	(1.33)	(1.21)	(1.01)	(1.14)	(1.12)	(1.20)	(1.22)
**Austria**	4.03	2.43	3.82	1.45	3.91	2.40	1.53	3.89	3.57	4.25	3.63	2.44	3.97	1.53	2.47
**N = 276**	(.98)	(1.06)	(1.03)	(.87)	(1.08)	(1.17)	(.77)	(1.05)	(1.09)	(.94)	(1.21)	(1.07)	(1.03	(.75)	(.98)
**Germany**	4.15	2.41	3.88	1.65	3.88	3.13	2.19	3.92	3.76	3.68	2.80	2.40	3.63	1.62	2.56
**N = 220**	(1.11)	(1.17)	(1.06)	(1.06)	(1.16)	(1.22)	(1.22)	(1.02)	(1.07)	(1.13)	(1.22)	(1.16)	(1.13	(.84)	(1.23)
**Poland**	4.26	2.15	3.70	2.07	4.30	4.08	2.12	3.56	3.62	2.88	2.83	1.95	2.85	1.96	2.04
**N = 163**	(.99)	(1.15)	(1.17)	(1.21)	(1.02)	(1.12)	(1.04)	(1.29)	(1.18)	(1.17)	(1.23)	(1.00)	(1.12)	(1.16)	(1.17)
**Hong Kong**	4.20	2.59	3.02	3.75	3.98	3.13	1.71	2.59	1.90	3.09	3.00	3.00	3.02	3.35	2.72
**N = 101**	(1.04)	(1.22)	(1.39)	(1.16)	(1.20)	(1.45)	(.95)	(1.50)	(1.17)	(1.21)	(1.17)	(1.29)	(1.26)	(1.22)	(1.38)
**India**	4.55	3.49	3.78	2.84	4.42	4.51	1.49	4.61	1.95	3.49	1.77	1.80	2.42	2.41	2.89
**N = 152**	(.51)	(.93)	(.73)	(.70)	(.54)	(.58)	(.55)	(.52)	(.77)	(.60)	(.70)	(.70)	(.69)	(.70)	(.63)
**USA**	3.97	3.19	3.11	3.34	3.98	3.28	2.38	3.11	2.95	2.92	2.30	2.37	2.79	2.61	2.89
**N = 184**	(1.25)	(1.34)	(1.31)	(1.43)	(1.20)	(1.40)	(1.24)	(1.35)	(.42)	(1.34)	(1.22)	(1.31)	(1.37)	(1.34)	(1.31)
**Norway**	4.17	2.74	4.16	1.94	4.27	3.24	2.38	3.86	3.88	3.98	2.72	3.52	3.72	3.24	2..95
**N = 187**	(1.11)	(1.27)	(.96)	(1.22)	(1.05)	(1.23)	(1.26)	(1.18)	(1.22)	(1.15)	(1.20)	(1.29)	(1.15)	(1.33)	(1.35)
**France**	4.66	3.01	3.57	3.81	3.82	3.14	1.57	3.19	4.13	2.45	2.51	2.31	2.58	1.76	2.58
**N = 216**	(.71)	(1.09)	(1.19)	(.97)	(1.16)	(1.33)	(.91)	(1.35)	(1.06)	(1.18)	(1.22)	(1.17)	(1.25)	(.96)	(1.29)
**Mexico**	4.63	2.60	3.80	2.53	4.07	3.48	2.01	3.81	2.77	2.55	2.97	2.32	3.12	2.16	2.41
**N = 146**	(.75)	(1.23)	(1.17)	(1.37)	(1.27)	(1.19)	(1.17)	(1.25	(1.31)	(1.20)	(1.30)	(1.21)	(1.12)	(1.15)	(1.04)
**Total**	4.24	2.71	3.78	2.50	4.07	3.27	2.07	3.57	3.07	3.39	2.62	2.51	3.31	2.28	2.56
**N = 2,009**	(1.03)	(1.23)	(1.14)	(1.50)	(1.13)	(1.31)	(1.16)	(1.27)	(1.36)	(1.28)	(1.29)	(1.24)	(1.22)	(1.20)	(1.22)
**Ranking**	1	9	3	13	2	7	15	4	8	5	10	12	6	14	11

Next, we looked at the four clusters of culturally resembling nations: Spain and Mexico; Germany, Austria, and The Netherlands; Great Britain and the United States, and Poland and the Czech Republic. Spain and Mexico differ significantly on only 4 (Spain scoring higher on justice, modesty and moderation, and Mexico on resolution) of the 15 virtues ratings. Germany also differs significantly on only 4 virtue ratings from Austria (Germany scoring higher on wisdom and moderation, and Austria higher on reliability, and resolution) and the Netherlands (The Netherlands scoring higher on reliability, faith, and mercy, and Germany higher on openness. Great Britain and the United States differ significantly on only 5 virtue ratings (Great Britain scoring higher on respect and helpfulness, and the United States on resolution, faith and moderation). Poland and the Czech Republic differ significantly on 7 virtue ratings. Apparently, the virtue ratings tend to be less different across nations that share a language, such as Spain and Mexico, Great Britain and the United States, or have a related language, as is the case with Germany and The Netherlands. France appears to differ from most nations. With Germany it differs significantly on 7 virtue ratings, and with Spain, another big European neighbor nation - in spite of a related linguistic and religious background - it differs significantly on 9 virtue ratings. Thus, France seems to have a specific more unique national virtue pattern. Norway differs from the Anglo-Saxon block on 7 virtue ratings from Great Britain and on 10 virtue ratings from the United States, respectively. Norway shows more resemblance with the Germanic block: it differs significantly on only 3 virtue ratings from Germany (Norway scoring higher on mercy, modesty and hope), and on 5 virtue ratings from the Netherlands, but it differs significantly on 8 virtue ratings from Austria.

### Relations of virtue ratings with age, gender and religion

No strong relations between age and importance ratings of virtues were found. One has to take into account that the respondents, being students, were on average relatively young. The highest – negative - relation (Spearman’s rho = −,152; *p* < .0001) found was with faith: the older the respondents the less importance they attributed to faith. The second highest (Spearman’s rho =,104; *p* < .0001) was with reliability: the older the respondents the more importance they attributed to reliability.

**Figure 3:**
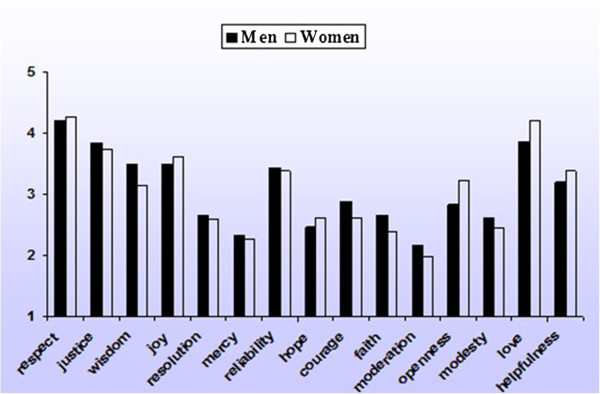
**Mean ratings of men (N = 1,049) and women (N = 1,721) on 15 virtues (1 = least important: 5 = most important).**

The rank order correlation (Spearman’s rho) between the overall male and female ratings was .94 (*p*<. 001). We did find significant differences between males and females (at the p<. 01 level) on nine virtue ratings, but they were relatively weak (the largest differences were found with respect to love, openness and wisdom; see Figure 3). Women attribute more importance to love (Partial eta squared = .022) and openness (Partial eta squared = .019) than men, but less to wisdom than men (Partial eta squared = .016). When we look at the national level, in most cases no significant gender differences on the importance ratings of virtues were found. The only exception is love. In 10 out of the 14 nations women find love significantly more important than men, testing with alpha = .01. No significant differences on love scores between males and females are found in Malaysia, the United States, France and Mexico.

**Figure 4:**
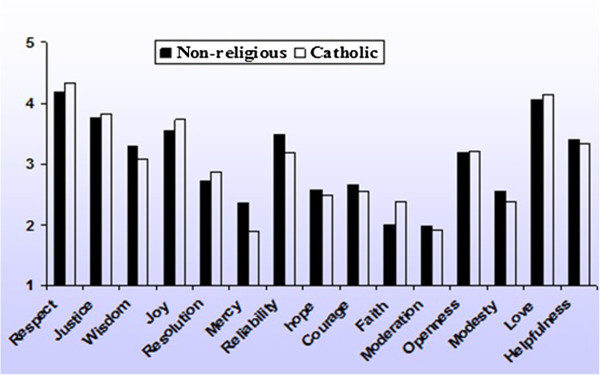
**Mean ratings of Nonreligious (N = 1,073) and Catholic respondents (N = 650) on 15 virtues (1 = least important: 5 = most important).**

Whereas a comparison between male and female respondents can be done because we had substantial numbers of male and female respondents from each nation, religion is strongly confounded with nation. The only realistic comparison regarding religion can be made between non-religious (N = 1073) and Catholic respondents (N = 650), because they form the largest groups and they are spread across all nations. As Figure 4 shows, differences between the two groups are in general very small. For obvious reasons non-religious respondents consider faith to be less important than Catholics. The only substantial other difference is mercy. Non-religious respondents rate it as more important as compared to Catholic respondents.

## Discussion

The present study examined the importance of virtues in contemporary societies. For that purpose, it used a combination of research methods: a free report questionnaire in which respondents could freely mention which good personal characteristics they would like to put into practice in their daily lives; and in addition they rank ordered a fixed list of 15 virtues that had been found to be important in a previous study in the Netherlands (Van Oudenhoven et al., 2012). Twelve of these 15 virtues were also freely mentioned as important virtues in the present study. The virtues hope and mercy were not frequently mentioned, but they resemble the frequently mentioned virtues of optimism and consideration, respectively. Only moderation was rarely freely mentioned, and it received the lowest score of all virtues on the importance ranking. Apparently the two methods lead to the conclusion that moderation is not a crucial virtue in contemporary societies. The combined results of the two approaches reinforce the validity of the conclusions.

Our main question was whether virtues differ between nations. They definitely do. Strong differences are found between nations both in the importance ratings of all virtues (the largest with respect to faith and the smallest with respect to love) and in the frequencies of the freely mentioned virtues respondents want to put into practice (honesty was frequently mentioned in all nations, but generosity only in France). The results are in accordance with an earlier study (Brezina and Van Oudenhoven 2012) on the conceptions of wisdom in five different nations (Ecuador, India, Indonesia, Malaysia and the United Arab Emirates) which showed that the influence of cross-national differences exceeded by far the influence of religion.

The second question “Are virtues shared by nations that show resemblance with respect to language, culture and/or religious composition” can also be positively answered as far as language is concerned. Differences in importance ratings between nations are smaller if nations share the same language: This holds for Great Britain versus the United States, Spain versus Mexico, and Austria versus Germany. The Netherlands, which has a language related to German, seems to belong to the Germanic cluster as well, although less convincingly than the other two. Germany and the Netherlands resemble each other more regarding religious composition than Austria and Germany, but Germany shows more overlap in virtues with Austria, with which it shares a language. Thus, the language seems to be more important than religion. This is in line with the earlier study (Brezina and Van Oudenhoven 2012), where also little evidence was found for the influence of religion. In general, the influence of religion appears to be small, as Figure 4 shows: Catholic and non-religious respondents tend to largely agree in their virtues ratings.

The third question was about the - relative - universality of virtues. Forty virtues were mentioned as important in at least one nation. On the basis of data from 14 nations we cannot draw strong conclusions on the universality of virtues. However, the results show that the freely mentioned virtues honesty (which is mentioned in all nations), respect (mentioned in 11 nations), kindness (mentioned in 10 nations), openness (mentioned in 9 nations), and tolerance (mentioned in 8 nations) are potential candidates. We may add love (mentioned in 5 nations) to that list because with a score of 4.07 it was rated as the second most important virtue after respect on the closed ended question. Moreover, there is relatively low variance in the love scores between the nations. Comparing these six contemporary virtues with the cardinal virtues Temperance (moderation), Prudence (wisdom), Fortitude (courage), Justice, Charity (love), Faith and Hope, we notice that the ‘modern’ virtues have a much stronger social character. Apparently, contemporary societies ask for virtues that contribute to good social relations.

Some virtues seem to be nation specific. Most notably the virtue ‘generosité’ (generosity) which was the most frequently mentioned virtue in France. Possibly the word ‘generosité’ may be interpreted differently than ‘generosity’ in other nations. Another nation specific virtue is ‘seguridad’ (certainty) which was not mentioned at all in any nation but Mexico. The importance of certainty for Mexicans is in accordance with the high preference of Mexico for avoiding uncertainty as indicated on Hofstede’s national culture dimensions (Hofstede et al. 2010). It has a score of 82 on the dimension of uncertainty avoidance on a scale of 1 – 120.

In contrast to the relatively large differences between national samples, there are only small differences in importance ratings of virtues between men and women, and between non-religious and Roman Catholic respondents. Apparently virtues are shared across categories within nations. These results suggest that people within a nation reinforce virtues in daily encounters. This may occur at schools, at home, at the work place, in the neighborhood, sport clubs, and other areas where people meet. A possible reason why virtues may be shared within nations is that they refer to values that are not ideological. They transcend values that are propagated by political parties or religious groups. Shared virtues facilitate intercultural or intergroup communication within a nation. That makes virtues a potentially powerful instrument to enhance cohesion within a nation between members from different cultural backgrounds.

## Electronic supplementary material

Additional file 1: **Questionnaire.** (DOC 37 KB)
