# Supplementary material for: Are virtues national, supranational, or universal?
Source: Springerplus. 2014 May 2;3:223. doi: 10.1186/2193-1801-3-223 (PMC4024483; doi:10.1186/2193-1801-3-223)
Supplement: Supplementary file 1 — Additional file 1: Questionnaire. (DOC 37 KB) [file 40064_2014_946_MOESM1_ESM.doc]

### Appendix 1: Questionnaire

### General

1. Gender:
2. Age:
3. Country of birth:
4. What is your mother tongue?

What is your religion/ philosophy of life?

- None
- I’m catholic
- I’m presbyterian
- I’m Lutheran
- I’m baptist
- I’m muslim
- I’m humanist
- Other,…………..

### What is your educational level?

### Primary school

- Lower vocational
- Middle vocational
- Lower general secondary
- Middle general secondary
- Higher professional education
- University
- Not applicable

1. What is your marital status?
2. What political party would you vote for?

**Specific questions:**

1. What do you find important personal characteristics which would you like to bring in practice in daily life?
2. What are bad personal characteristics to you?
3. Which characteristics may, to your opinion, improve relations between different cultural groups in your country of origin?

12. Below are 15 personal characteristics. We would like to ask you to place **all** 15 characteristics in the table. Put the 3 characteristics that you find most important in the first column, next the 3 characteristics that you then find most important in the second column, etcetera.

**respect
courage**

**justice
faith
 wisdom
 moderation**

**joy
 openness**

**resolution**

**modesty
helpfulness
mercy**

| ***Most important*** ***Least important***  **Column 1 Column 2 Column 3 Column 4 Column 5** | | | | |
| --- | --- | --- | --- | --- |
|  |  |  |  |  |
|  |  |  |  |  |
|  |  |  |  |  |

**love reliability hope**
